# Supplementary material for: Profile, Infection, and Vaccination Uptake: A Cohort of Canadian Retail Workers During the SARS-CoV-2 Pandemic
Source: Infect Dis Rep. 2025 Sep 29;17(5):122. doi: 10.3390/idr17050122 (PMC12564636; doi:10.3390/idr17050122)
Supplement: Supplementary file 1 [file idr-17-00122-s001.zip › idr-3823132-supplementary.pdf]

Table S1. Study procedures

| | Visit 1 N=304 | Visit 2 N=297 | Visit 3 N=291 | Visit 4 N=198 | Visit 5 N=194 | COVID-19 visit (VCoV) N=15 |
|---|---|---|---|---|---|---|
| Retention | 100% | 98% | 96% | 100% | 98% | - |
| Eligibility assessment | X | | | X | | |
| Consent form | X | | | X | | |
| **Participant characteristics** | | | | | | |
| Demographic data | X | | | | | |
| Household data | X | | | | | |
| Clinical data | X | | | | | |
| Occupational data | X | X | X | X | X | X |
| **Retrospective questionnaire form** | | | | | | |
| Influenza vaccine Season 2020-2021 | X | | | | | |
| COVID-19 symptoms | X | X | X | X | X | X |
| Positive SARS-CoV-2 detection tests | X | X | X | X | X | X |
| SARS-CoV-2 vaccine | X | X | X | X | X | X |
| **Cross-section interventions** | | | | | | |
| Humoral immunity blood samples | X | X | X | X | X | X |
| Cellular immunity blood samples | X | | X | | X | X |
| SARS-CoV-2 PCR test at visit | | | | X | X | |

Table S2. Household characteristics of the participants at the first visit

| | **Overall study population** | | **Restaurant/bar workers** | | **Grocery store workers** | | **Hardware store workers** | |
|---|---|---|---|---|---|---|---|---|
| | **Total (N=304)** | **COVID-19[1] (N=117)** | **Total (N=149)** | **COVID-19[1] (N=62)** | **Total (N=112)** | **COVID-19[1] (N=42)** | **Total (N=43)** | **COVID-19[1] (N=13)** |
| **Region, N (%)** | | | | | | | | |
| Capitale-Nationale | 231 (76.0%) | 91 (77.8%) | 112 (75.2%) | 45 (72.6%) | 86 (76.8%) | 35 (83.3%) | 33 (76.7%) | 11 (84.6%) |
| Chaudière-Appalaches | 73 (24.0%) | 26 (22.2%) | 37 (24.8%) | 17 (27.4%) | 26 (23.2%) | 7 (16.7%) | 10 (23.3%) | 2 (15.4%) |
| **Household size, N (%)** | | | | | | | | |
| 1-2 residents | 188 (61.8%) | 76 (65.0%) | 99 (67.1%) | 48 (77.4%) | 57 (50.9%) | 19 (45.2%) | 32 (74.4%) | 9 (69.2%) |
| 3-4 residents | 94 (30.9%) | 32 (27.4%) | 36 (24.2%) | 10 (16.1%) | 48 (42.9%) | 19 (45.2%) | 9 (20.9%) | 3 (23.1%) |
| ≥5 residents | 22 (7.2%) | 9 (7.7%) | 13 (8.7%) | 4 (6.5%) | 7 (6.3%) | 4 (9.5%) | 2 (4.7%) | 1 (7.7%) |
| **Co-residents, N (%)** | | | | | | | | |
| Children (<18) | 70 (23.0%) | 29 (24.8%) | 35 (23.5%) | 12 (19.4%) | 29 (25.9%) | 14 (33.3%) | 6 (14.0%) | 3 (23.1%) |
| Healthcare worker | 47 (15.5%) | 17 (14.5%) | 23 (15.4%) | 9 (14.5%) | 18 (16.1%) | 5 (11.9%) | 6 (14.0%) | 3 (23.1%) |
| Kindergarden worker | 10 (3.3%) | 3 (2.6%) | 5 (3.4%) | 1 (1.6%) | 5 (4.5%) | 2 (4.8%) | 0 (0.0%) | 0 (0.0%) |
| Teacher | 13 (4.3%) | 7 (6.0%) | 9 (6.0%) | 4 (6.5%) | 4 (3.6%) | 3 (7.1%) | 0 (0.0%) | 0 (0.0%) |
| **Household bedroom, N (%)** | | | | | | | | |
| 1-2 | 158 (52.0%) | 66 (56.4%) | 90 (60.4%) | 41 (66.1%) | 49 (43.8%) | 19 (45.2%) | 19 (44.2%) | 6 (46.2%) |
| ≥3 | 146 (48.0%) | 51 (43.6%) | 59 (39.6%) | 21 (33.9%) | 63 (56.3%) | 23 (54.8%) | 24 (55.8%) | 7 (53.8%) |

1. Subset of participants who reported a positive SARS-CoV-2 test (PCR or rapid antigen) at least once during the study period.

Table S3. Protective measures taken at work and elsewhere by study participants recorded at first visit

| | Overall study population | | Restaurant/bar workers | | Grocery store workers | | Hardware store workers | |
|---|---|---|---|---|---|---|---|---|
| | Total (N=304) | COVID-19[1] (N=117) | Total (N=149) | COVID-19[1] (N=62) | Total (N=112) | COVID-19[1] (N=42) | Total (N=43) | COVID-19[1] (N=13) |
| **Protection measures at work,[2] N (%)** | | | | | | | | |
| Mask | 299 (98.4%) | 116 (99.1%) | 147 (98.7%) | 61 (98.4%) | 111 (99.1%) | 42 (100.0%) | 41 (95.3%) | 13 (100.0%) |
| Handwashing | 299 (98.4%) | 114 (97.4%) | 145 (97.3%) | 59 (95.2%) | 111 (99.1%) | 42 (100.0%) | 43 (100.0%) | 13 (100.0%) |
| Plexiglas | 235 (77.3%) | 88 (75.2%) | 96 (64.4%) | 39 (62.9%) | 98 (87.5%) | 37 (88.1%) | 41 (95.3%) | 12 (92.3%) |
| Social distancing | 213 (70.1%) | 86 (73.5%) | 111 (74.5%) | 48 (77.4%) | 74 (66.1%) | 29 (69.0%) | 28 (65.1%) | 9 (69.2%) |
| Protective glasses | 77 (25.3%) | 12 (10.3%) | 30 (20.1%) | 4 (6.5%) | 35 (31.3%) | 5 (11.9%) | 12 (27.9%) | 3 (23.1%) |
| Faceshield | 32 (10.5%) | 4 (3.4%) | 16 (10.7%) | 1 (1.6%) | 13 (11.6%) | 2 (4.8%) | 3 (7.0%) | 1 (7.7%) |
| Gloves | 21 (6.9%) | 3 (2.6%) | 10 (6.7%) | 0 (0.0%) | 9 (8.0%) | 3 (7.1%) | 2 (4.7%) | 0 (0.0%) |
| Face Cover | 7 (2.3%) | 1 (0.9%) | 3 (2.0%) | 0 (0.0%) | 4 (3.6%) | 1 (2.4%) | 0 - | 0 (0.0%) |
| Other[3] | 135 (44.4%) | 59 (50.4%) | 85 (57.0%) | 41 (66.1%) | 43 (38.4%) | 16 (38.1%) | 7 (16.3%) | 2 (15.4%) |
| **Behavioral protection measures,[2] N (%)** | | | | | | | | |
| Mask wearing in public spaces | 304 (100.0%) | 117 (100.0%) | 149 (100.0%) | 62 (100.0%) | 112 (100.0%) | 42 (100.0%) | 43 (100.0%) | 12 (92.3%) |
| Avoid usual salutations | 259 (85.2%) | 99 (84.6%) | 119 (79.9%) | 48 (77.4%) | 103 (92.0%) | 39 (92.9%) | 37 (86.0%) | 12 (92.3%) |
| Social distancing | 256 (84.2%) | 93 (79.5%) | 115 (77.2%) | 41 (66.1%) | 102 (91.1%) | 40 (95.2%) | 39 (90.7%) | 12 (92.3%) |
| Avoid contacts with vulnerable persons | 254 (83.6%) | 93 (79.5%) | 114 (76.5%) | 42 (67.7%) | 100 (89.3%) | 38 (90.5%) | 40 (93.0%) | 13 (100.0%) |
| Avoid crowded places | 233 (76.6%) | 81 (69.2%) | 102 (68.5%) | 35 (56.5%) | 94 (83.9%) | 34 (81.0%) | 37 (86.0%) | 12 (92.3%) |
| Quarantine if exposed to COVID-19 | 126 (41.4%) | 70 (59.8%) | 65 (43.6%) | 36 (58.1%) | 46 (41.1%) | 25 (59.5%) | 15 (34.9%) | 9 (69.2%) |
| Pre-emptive isolation | 36 (11.8%) | 23 (19.7%) | 21 (14.1%) | 13 (21.0%) | 12 (10.7%) | 9 (21.4%) | 3 (7.0%) | 1 (7.7%) |
| **Handwashing habits,[2] N (%)** | | | | | | | | |
| After using the restroom | 297 (97.7%) | 115 (98.3%) | 147 (98.7%) | 60 (96.8%) | 109 (97.3%) | 42 (100.0%) | 41 (95.3%) | 13 (100.0%) |
| When dirty | 294 (96.7%) | 113 (96.6%) | 140 (94.0%) | 58 (93.5%) | 112 (100.0%) | 42 (100.0%) | 42 (97.7%) | 13 (100.0%) |
| When entering workspace | 280 (92.1%) | 106 (90.6%) | 136 (91.3%) | 55 (88.7%) | 107 (95.5%) | 37 (88.1%) | 37 (86.0%) | 13 (100.0%) |
| Before eating | 267 (87.8%) | 104 (88.9%) | 124 (83.2%) | 51 (82.3%) | 105 (93.8%) | 41 (97.6%) | 38 (88.4%) | 12 (92.3%) |
| Before & after handling food | 246 (80.9%) | 96 (82.1%) | 127 (85.2%) | 55 (88.7%) | 93 (83.0%) | 32 (76.2%) | 26 (60.5%) | 9 (69.2%) |
| After handling trash | 242 (79.6%) | 97 (82.9%) | 119 (79.9%) | 51 (82.3%) | 95 (84.8%) | 38 (90.5%) | 28 (65.1%) | 8 (61.5%) |
| When exiting workspace | 218 (71.7%) | 81 (69.2%) | 104 (69.8%) | 41 (66.1%) | 87 (77.7%) | 32 (76.2%) | 27 (62.8%) | 8 (61.5%) |
| Other[3] | 50 (16.4%) | 11 (9.4%) | 29 (19.5%) | 4 (6.5%) | 12 (10.7%) | 2 (4.8%) | 9 (20.9%) | 5 (38.5%) |

1. Subset of participants who reported a positive SARS-CoV-2 test (PCR or rapid antigen) at least once during the study period.

2. Includes customer registry, QR code, customer limit in store, thorough cleaning of workplace, worker temperature surveillance.

3. Includes after touching the cash register, handling money, in between clients
